# Polymorphisms of cytokine genes and tuberculosis in two independent studies

**DOI:** 10.1038/s41598-019-39249-4

**Published:** 2019-02-21

**Authors:** Shouquan Wu, Ming-Gui Wang, Yu Wang, Jian-Qing He

**Affiliations:** Department of Respiratory and Critical Care Medicine, West China Hospital, Sichuan University, Chengdu, Sichuan China

## Abstract

Cytokine gene single nucleotide polymorphisms (SNPs) can influence cytokine levels, which may be associated with tuberculosis (TB) susceptibility. There is evidence that interleukin 1B (IL1B), tumor necrosis factor-alpha (TNF-alpha), and IL6 may be involved in the progression of TB. Using a self-validating case-control design, we selected eleven functional SNPs in *IL1B*, *TNF* and *IL6* to detect their association with TB in Chinese Han and Tibetan populations. The associations between SNPs and TB were estimated by computing the odds ratios (ORs) and 95% confidence intervals (95% CI) using logistic regression analyses. We found that the *IL1B* rs16944 polymorphism was associated with decreased risk of TB in the two studies. The G allele at rs2069837 of *IL6* was significantly more common in controls than in TB patients in the Han population. Moreover, *TNF* rs1799964 and rs1800630 were risk factors for susceptibility to TB, which were validated in the Chinese Tibetan population. In addition, *TNF* rs1799724 and rs1800629 were associated with TB, but only in the Tibetan population. In conclusion, SNPs of the *IL1B* and *TNF* gene were associated with TB susceptibility in Chinese Han and Tibetan populations. *IL6* polymorphism may be considered as a protective factor for TB in the Chinese Han population, but not the Tibetan population.

## Introduction

Tuberculosis (TB) is an ancient infectious disease caused by *Mycobacterium tuberculosis* (*M*. *TB*) which can spread not only to the respiratory system but also to other organs. In 2017, the World Health Organization (WHO) reported that the incidence of TB in China was 64 per 100,000 individuals^1^. China has ranked the number three regarding the number of TB patients and the Tibetan nationality in this country has the highest incidence rate^2^. An estimated one-third of the world’s population is infected with *M*. *TB*, but only 5–15% of infected individuals develop active TB and the rest remain asymptomatic^3^. The reason why only a minority of the infected individuals develops TB disease is still largely unknown. It was suggested that the interactions between the bacterial agent, environmental and genetic factors play important roles in the progression to TB disease^4^.

Studies of concordance of disease in twins have shown that host genetic factors play significant roles in TB disease^5,6^. Determining the specific host genes associated with TB disease may enhance the understanding of the pathogenesis of TB and further the development of treatment strategies. To date, many studies have suggested that cytokine gene polymorphisms are associated with TB among different populations. For example, interferon gamma (*IFNG*) and interleukin 17 (*IL17*) polymorphisms were reported to be associated with TB risk^7,8^. Several meta-analyses suggested that cytokines such as IL10, IL12B, IL27 and CC chemokine ligand 5 (CCL5) play important roles in the progression of TB^9–12^. Indeed, genetic variants in cytokine genes could affect the recognition sites of the transcription factors, leading to altered transcriptional activity, which may then result in a change in cytokine production levels^13^. Intricate interplay among lymphocytes, antigen-presenting cells and released cytokines is involved in the immune response against TB.

Polymorphisms in the *IL1B*, tumor necrosis factor (*TNF*), and *IL6* genes have been associated with susceptibility to TB in previous studies. The *IL1B* gene is located on chromosome 2. IL1B is a major proinflammatory cytokine that is produced by monocytes, macrophages and dendritic cells during infection and inflammation. *IL1B* gene polymorphisms have been demonstrated alter IL1B protein production^14,15^. IL1B plays a critical role in the immune response to mycobacteria, and may increase resistance to initial infection. *IL1B* polymorphisms has been associated with TB in human^16^. TNF-alpha is a multifunctional cytokine that is synthesized by monocytes, macrophages, neutrophils, T-cells, and natural killer-cells^17^. These cells have been described to play a critical role in the immunopathogenesis of TB. TNF-alpha also participates in the granulomatous reaction which could further limit *M*. *TB* growth. Single nucleotide polymorphisms (SNPs) within *TNF* were suggested substantially to affect TNF-alpha production levels^18^. Several studies have shown a significant association between *TNF* polymorphisms and TB^19,20^. IL6 is produced by activated monocytes and macrophages. It is an important immunoregulatory factor, which can reduce the production of IL1B and TNF-alpha^21^. Previous studies have shown that TB patients had higher IL6 levels, compared with healthy controls^22^. Also, it could increase early IFN-γ secretion in MTB infection and thus help to control TB infection^23^. IL6-deficient mice were more susceptible to TB than wild-type mice^24^ and polymorphisms in *IL6* were reported to be associated with TB^25^.

Despite the wealth of association studies of *IL1B*, *TNF* and *IL6* polymorphisms a consensus has yet to emerge as to which variants affect susceptibility to TB. In addition, none of the previous studies was conducted with a validation cohort. Thus, we hypothesized that *ILB*, *IL6* and *TNF* polymorphisms are associated with TB in Chinese Han and Tibetan populations, and performed two independent studies with TB cases and healthy controls in these two populations to determine the influence of the three cytokine gene polymorphisms on TB susceptibility.

## Results

### Demographics of the study population

In the initial cohort, we recruited 636 TB cases (50.9% males) and 608 controls (49.7% males) from the Chinese Han population. The mean (±SD) age was 36.8 (±15.7) years for cases and 37.1 (±15.7) years for controls. There were no significant differences in sex and age between groups (Table 1). The percentage of smoking is lower in cases than in controls (*P* = 0.003) (Table 1). In the validation cohort, 613 TB patients (53.3% males; mean (±SD) age was 34.5 (±14.5) years) and 603 healthy subjects (55.2% males; mean (±SD) age was 34.6 (±13.8) years) were enrolled from the Tibetan population. Cases and controls in this group were also well-matched by age and gender (Table 1). However, data of smoking status among the Tibetan subjects was not collected because smoking rates were very low in this population for cultural reasons.

**Table 1:** Demographic distribution of healthy controls and tuberculosis patients.

Parameters	Cases	Controls	*P* value
Han population	n = 636	n = 608	
Age (mean ± SD, years)	36.8 ± 15.7	37.1 ± 15.7	0.677
Male, n (%)	324 (50.9)	302 (49.7)	0.654
Smoking, n (%)	195 (69.3)	141 (76.8)	0.003?
Location of TB, n (PTB/EPTB)	276/360		
Acid-fast bacilli stain positive, n (positive/negative)	138/360		
Culture positive n (positive/negative)	32/126		
TB-DNA positive n (positive/negative)	122/133		
Tibetan population	n = 613	n = 603	
Age, (mean ± SD, years)	34.5 ± 14.5	34.6 ± 13.8	0.909
Male, n (%)	327 (53.3)	333 (55.2)	0.511

Abbreviations: SD, standard deviation; PTB, pulmonary tuberculosis; EPTB, extra-pulmonary tuberculosis.

### Association between SNPs and TB susceptibility in two studies

Eleven SNPs previously investigated in genetic studies of TB in the *IL1B*, *TNF* and *IL6* genes were selected for this study (Table S1 and Table S2). Among the 11 SNPs, rs1143623^26^, rs2069837^27^, rs1799724^28^, rs1800629^28^, and rs1800630^28^ were associated with transcriptional activity of the genes. rs361525^29^, rs1799724^30^, rs1800630^31^, rs1800629^32^, rs1799964^31^, rs1143634^33^, rs16944^34^, and rs1800795^35^ polymorphisms may influence protein levels of their respective cytokines. rs1800630^36^, rs1799724^36^, rs1800795^37^, rs16944^38^, rs1800629^38^, rs1799964^39^ and rs361525^40^ were contributory factors to *TNF* gene expression. rs17147230 was predicted to have functional significance by the FuncPred algorithm. Only rs1800630 deviated from HWE and only in the Tibetan control subjects (*P* = 0.024). The characteristics of the SNPs are shown in Table 2.

**Table 2:** Characteristics of the SNPs in the study.

Gene/SNPs	Chr.	SNP	Location in gene	Function*	MA	MAF	MA	MAF	HWE	
					Han	Han	Tibetan	Tibetan	Han	Tibetan
*IL1B*	2	rs1143634	exon5 (nonsynonymous)	IL1B levels	A	0.02	A	0.03	0.340	0.776
		rs16944	5′FLANKING	*IL1B* expression, IL1B levels	A	0.48	G	0.42	0.999	0.976
		rs1143623	5′FLANKING	TFBS	G	0.41	G	0.48	0.731	0.991
*IL6*	7	rs17147230	Unkonwn	TFBS	T	0.44	A	0.48	0.645	0.554
		rs1800795	5′FLANKING	*IL6* expression, IL6 levels	C	0.002	C	0.002	0.993	0.999
		rs2069837	intron2	TFBS	C	0.17	C	0.26	0.366	0.118
*TNF*	6	rs1799964	5′FLANKING	*TNF* expression, TNF levels	C	0.17	C	0.22	0.986	0.075
		rs1800630	5′FLANKING	*TNF* expression, TFBS, TNF-alpha levels	A	0.16	A	0.18	0.841	0.024
		rs1799724	5′FLANKING	*TNF* expression, TFBS, TNF-alpha levels	C	0.14	C	0.16	0.951	0.989
		rs1800629	5′FLANKING	*TNF* expression, TNF-alpha levels	A	0.07	A	0.02	0.869	0.092
		rs361525	5′FLANKING	*TNF* expression, TNF-alpha levels	A	0.02	A	0.04	0.281	0.686

Abbreviation: SNP, single nucleotide polymorphism; MA, minor allele; MAF, minor allele frequency; HWE, Hardy Weinberg equilibrium; TFBS, transcription factor binding sites; Chr. chromosome.

*Function of each SNP was reported by previous studies and/or predicted from the NIH FuncPred website (https://snpinfo.niehs.nih.gov/snpinfo/snpfunc.html).

**Figure 1:**
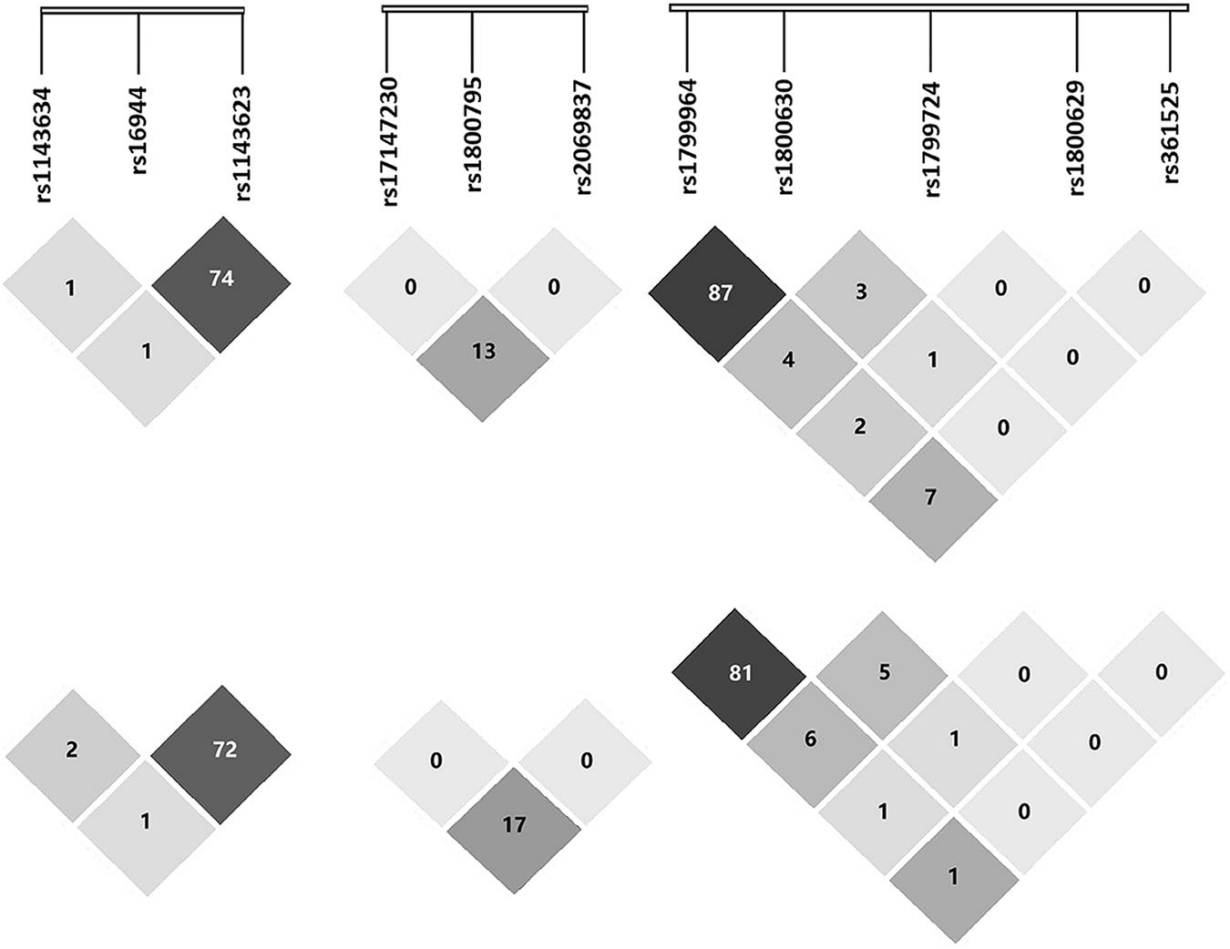
Linkage disequilibrium (LD) of cytokine gene SNPs in the both Han (above) and Tibetan (below) populations. LD r2 values (ranging from 0 to 1) for all pairs of SNPs are presented as percentages. Shading from white to black indicates the extent of LD measured as r2.

We observed that the three gene polymorphisms were associated with TB (Tables 3 and 4). In *IL1B*, at the rs1143634 polymorphic site, the AA + GA genotypes compared with GG were protective factors against TB (*P* = 0.042). *In IL6*, the rs2069837 G allele (*P* = 0.046) and the G carriers (GG + GA) (*P* = 0.044) were associated with decreased risk of TB. In *TNF*, two SNPs were significantly associated with TB under the dominant model, i.e., rs1799964 (*P* = 0.017) and rs1800630 (*P* = 0.002). We also determined that CT heterozygotes and the C allele of rs1799964 were risk factors for susceptibility to TB. In addition, CA heterozygotes (*P* = 0.024) and the A allele (*P* = 0.020) of rs1800630 occurred at a higher frequency in the TB cases than in the control group. The LD between the SNPs is shown in Fig. 1. We found that the *TNF* frequency of haplotype CACGG was significantly different between TB and control groups (Table 5).

**Table 3:** Genotype distribution of cytokine genes in the two populations.

Gene/SNPs			Han population				Tibetan population			
			Case(%), n = 636	Control(%), n = 608	*P* ^#^	OR^#^(95% CI)	Case(%), n = 613	Control(%), n = 603	*P* ^#^	OR^#^(95% CI)
*IL1B*	rs1143634	Genotype								
		GG	604 (95.6)	288 (94.4)			566 (92.3)	568 (94.4)		
		GA	28 (4.4)	16 (5.2)	0.094	1.71 (0.91–3.19)	46 (7.5)	34 (5.6)	0.184	1.37 (0.86–2.16)
		AA	0 (0)	1 (0.3)	—		1 (0.2)	0 (0.0)	—	
		Allele								
		G	1236 (97.8)	592 (97.0)			1178 (96.1)	1170 (97.2)		
		A	28 (2.2)	18 (3.0)	0.183	1.50 (0.83–2.73)	48 (3.9)	34 (2.8)	0.133	1.41 (0.90–2.21)
	rs16944	Genotype								
		GG	164 (25.9)	164 (27.1)			110 (17.9)	131 (21.7)	0.028	0.69 (0.50–0.96)
		GA	309 (48.9)	302 (49.9)	0.463	0.90 (0.68–1.19)	298 (48.6)	303 (50.2)	0.105	0.81 (0.62–1.05)
		AA	159 (25.2)	139 (23.0)	0.413	0.88 (0.64–1.20)	205 (33.4)	169 (28.0)		
		Allele								
		G	637 (50.4)	630 (52.1)			518 (42.3)	565 (46.8)	0.023	0.83 (0.71–0.97)
		A	627 (49.6)	580 (47.9)	0.422	1.07 (0.91–1.25)	708 (57.7)	641 (53.2)		
	rs1143623	Genotype								
		CC	198 (31.3)	203 (33.6)			155 (25.3)	162 (26.9)		
		GC	309 (48.9)	303 (50.1)	0.709	1.05 (0.82–1.35)	294 (48.0)	299 (49.7)	0.731	1.05 (0.80–1.38)
		GG	125 (19.8)	99 (16.4)	0.132	1.29 (0.93–1.79)	164 (26.8)	141 (23.4)	0.226	1.22 (0.89–1.67)
		Allele								
		C	705 (55.8)	709 (58.6)			604 (49.3)	623 (51.7)		
		G	559 (44.2)	501 (41.4)	0.163	1.12 (0.96–1.31)	622 (50.7)	581 (48.3)	0.222	1.10 (0.94–1.30)
*IL6*	rs17147230	Genotype								
		AA	197 (31.2)	183 (30.2)			123 (20.1)	143 (23.8)	0.290	0.84 (0.61–1.16)
		AT	315 (49.8)	289 (47.8)	0.214	1.21 (0.90–1.62)	313 (51.1)	287 (47.7)	0.665	1.06 (0.81–1.38)
		TT	120 (19.0)	133 (22.0)	0.273	1.20 (0.87–1.65)	177 (28.9)	172 (28.6)		
		Allele								
		A	709 (56.1)	655 (54.1)			559 (45.6)	573 (47.6)	0.344	0.93 (0.79–1.09)
		T	555 (43.9)	555 (45.9)	0.328	0.92 (0.79–1.08)	667 (54.4)	631 (52.4)		
	rs1800795	Genotype								
		GG	630 (99.7)	599 (99.0)			606 (98.9)	601 (99.7)		
		GC	2 (0.3)	6 (1.0)	0.161	0.32 (0.06–1.58)	6 (1.0)	2 (0.3)	0.185	2.96 (0.60–14.74)
		CC	0 (0)	0 (0)	—		1 (0.2)	0 (0)	—	
		Allele								
		G	1262 (99.8)	1204 (99.5)			1218 (99.3)	1204 (99.8)		
		C	2 (0.2)	6 (0.5)	0.161	0.32 (0.06–1.58)	8 (0.7)	2 (0.2)	0.082	3.97 (0.84–18.74)
	rs2069837	Genotype								
		AA	443 (70.1)	392 (64.8)			340 (55.5)	338 (56.1)		
		GA	163 (25.8)	183 (30.2)	0.060	0.79 (0.61–1.01)	226 (36.9)	213 (35.4)	0.674	1.05 (0.83–1.34)
		GG	26 (4.1)	30 (5.0)	0.328	0.76 (0.44–1.31)	47 (7.7)	51 (8.5)	0.697	0.92 (0.60–1.41)
		Allele								
		A	1049 (83.0)	967 (79.9)			906 (73.9)	889 (73.8)		
		G	215 (17.0)	243 (20.1)	0.046	0.81 (0.66–0.99)	320 (26.1)	315 (26.2)	0.972	0.99 (0.83–1.20)
*TNF*	rs1799964	Genotype								
		TT	390 (61.7)	413 (68.3)			329 (53.7)	372 (61.8)		
		CT	212 (33.5)	173 (28.6)	0.037	1.30 (0.02–1.66)	239 (39.0)	190 (31.6)	0.004	1.43 (1.12–1.82)
		CC	30 (4.7)	19 (3.1)	0.099	1.65 (0.91–2.98)	45 (7.3)	40 (6.6)	0.286	1.28 (0.81–2.01)
		Allele								
		T	992 (78.5)	999 (82.6)			897 (73.2)	934 (77.6)		
		C	272 (21.5)	211 (17.4)	0.011	1.30 (1.06–1.58)	329 (26.8)	270 (22.4)	0.011	1.27 (1.06–1.53)
	rs1800630	Genotype								
		CC	410 (64.9)	431 (71.2)			372 (60.7)	417 (69.2)		
		CA	199 (31.5)	157 (26.0)	0.024	1.33 (1.04–1.71)	208 (33.9)	157 (26.0)	0.002	1.49 (1.16–1.92)
		AA	23 (3.6)	17 (2.8)	0.312	1.39 (0.73–2.65)	33 (5.4)	29 (4.8)	0.359	1.28 (0.76–2.14)
		Allele								
		C	1019 (80.6)	1019 (84.2)			952 (77.7)	991 (82.2)		
		A	245 (19.4)	191 (15.8)	0.020	1.28 (1.04–1.58)	274 (22.3)	215 (17.8)	0.005	1.33 (1.09–1.62)
	rs1799724	Genotype								
		CC	462 (73.1)	436 (72.1)			368 (60.0)	430 (71.3)		
		CT	160 (25.3)	154 (25.5)	0.889	0.98 (0.76–1.27)	206 (33.6)	157 (26.0)	0.001	1.53 (1.19–1.97)
		TT	10 (1.6)	15 (2.5)	0.257	0.63 (0.28–1.41)	39 (6.4)	16 (2.7)	0.001	2.87 (1.58–5.22)
		Allele								
		T	1084 (85.8)	1026 (84.8)			942 (76.8)	1017 (84.3)		
		C	180 (14.2)	184 (15.2)	0.499	0.93 (0.74–1.16)	284 (23.2)	189 (15.7)	<0.001	1.62 (1.32–1.99)
	rs1800629	Genotype								
		GG	541 (85.6)	510 (84.3)			587 (95.8)	560 (93.0)		
		GA	88 (13.9)	92 (15.2)	0.526	0.90 (0.66–1.24)	25 (4.1)	42 (7.0)	0.028	0.57 (0.34–0.94)
		AA	3 (0.5)	3 (0.5)	0.954	0.95 (1.91–4.75)	1 (0.2)	0 (0)	—	
		Allele								
		G	1170 (92.6)	1112 (91.9)			1199 (97.8)	1162 (96.5)		
		A	94 (7.4)	98 (8.1)	0.545	0.91 (0.68–1.23)	27 (2.2)	42 (3.5)	0.056	0.62 (0.38–1.01)
	rs361525	Genotype								
		GG	607 (96.0)	582 (96.2)			559 (91.2)	553 (91.7)		
		GA	24 (3.8)	22 (3.6)	0.892	1.04 (0.58–1.88)	52 (8.5)	48 (8.0)	0.718	1.08 (0.72–1.63)
		AA	1 (0.2)	1 (0.2)	0.984	0.97 (0.06–15.64)	2 (0.3)	2 (0.3)	0.989	0.99 (0.14–7.04)
		Allele								
		G	1238 (97.9)	1186 (98.0)			1170 (95.4)	1154 (95.7)		
		A	26 (2.1)	24 (2.0)	0.908	1.03 (0.59–1.81)	56 (4.6)	52 (4.3)	0.740	1.07 (0.73–1.57)

Abbreviation: SNPs, single nucleotide polymorphisms; CI, confidence interval; OR, odds ratio.

^#^Adjusted by age and sex status.

**Table 4:** Association between genotype of cytokine genes and TB in the two populations.

Gene/SNPs	Han population		Tibetan population	
	*P* ^#^	OR^#^ (95% CI)	*P* ^#^	OR^#^ (95% CI)
*IL1B*				
**rs1143634**				
Dominant model	0.130	1.61 (0.87–2.97)	0.154	1.40 (0.88–2.21)
Recessive model	—		—	
**rs16944**				
Dominant model	0.042	0.78 (0.61–0.99)	0.383	0.89 (0.69–1.56)
Recessive model	0.098	0.79 (0.59–1.05)	0.660	0.95 (0.73–1.22)
**rs1143623**				
Dominant model	0.414	1.11 (0.87–1.40)	0.508	1.09 (0.84–1.41)
Recessive model	0.123	1.26 (0.94–1.68)	0.186	1.19 (0.92–1.55)
*IL6*				
**rs17147230**				
Dominant model	0.193	1.20 (0.91–1.59)	0.934	0.99 (0.77–1.27)
Recessive model	0.725	1.04 (0.82–1.33)	0.128	0.81 (0.62–1.06)
**rs1800795**				
Dominant model	0.161	0.32 (0.06–1.58)	0.121	3.47 (0.72–16.79)
Recessive model	—		—	
**rs2069837**				
Dominant model	0.044	0.78 (0.62–0.99)	0.820	1.03 (0.82–1.29)
Recessive model	0.462	0.82 (0.48–1.40)	0.621	0.90 (0.60–1.36)
*TNF*				
**rs1799964**				
Dominant model	0.017	1.33 (1.05–1.68)	0.004	1.40 (1.11–1.76)
Recessive model	0.158	1.53 (0.85–2.74)	0.627	1.12 (0.72–1.74)
**rs1800630**				
Dominant model	0.002	1.46 (1.15–1.85)	0.017	1.34 (1.05–1.70)
Recessive model	0.652	1.13 (0.67–1.88)	0.421	1.30 (0.69–2.46)
**rs1799724**				
Dominant model	0.686	0.95 (0.74–1.22)	<0.001	1.65 (1.30–2.10)
Recessive model	0.263	0.63 (0.28–1.41)	0.003	2.49 (1.37–4.50)
**rs1800629**				
Dominant model	0.528	0.90 (0.66–1.24)	0.038	0.59 (0.36–0.97)
Recessive model	0.964	0.96 (0.19–4.80)	—	
**rs361525**				
Dominant model	0.899	1.04 (0.58–1.85)	0.727	1.07 (0.72–1.61)
Recessive model	0.973	0.95 (0.06–15.31)	0.982	0.98 (0.14–6.97)

Abbreviation: SNP, single nucleotide polymorphism; CI, confidence interval; OR, odds ratio.

^#^Adjusted by sex and age.

**Table 5:** Haplotypes of the cytokines genes and their distributions in the two cohorts.

Gene/haplotype	Han population				Tibetan population			
	Case(%) n = 1264	Control(%) n = 1210	P	OR	Case(%) n = 1226	Control(%) n = 1210	P	OR
***IL1B***								
AGC	—				46.4 (3.8)	16.62 (1.4)	<0.001	2.82 (1.60–4.96)
GAC	75.4 (6.0)	86.6 (7.2)	0.251	0.83 (0.60–1.42)	91.4 (7.5)	86.6 (7.2)	0.785	1.04 (0.77–1.42)
GAG	551.5 (43.6)	492.1 (40.7)	0.097	1.15 (0.98–1.35)	615.0 (50.2)	492.1 (40.7)	<0.001	1.47 (1.25–1.72)
GGC	601.6 (47.6)	605.8 (50.1)	0.285	0.92 (0.78–1.08)	466.3 (38.0)	605.8 (50.1)	<0.001	0.61 (0.52–0.72)
Other^*^	35.4 (2.8)	25.6 (2.1)	?	?	7.0 (0.5)	8.9 (0.7)	?	?
***IL6***								
AGA	674.3 (53.3)	615.9 (50.9)	0.259	1.01 (0.94–1.28)	531.0 (43.3)	615.9 (50.9)	<0.001	0.74 (0.63–0.87)
AGG	34.73 (2.7)	39.11 (3.2)	0.470	0.84 (0.53–1.34)	26.62 (2.2)	39.1 (3.2)	0.108	0.67 (0.40–1.10)
TGA	372.8 (29.5)	345.1 (22.7)	0.633	1.04 (0.88–1.24)	367.0 (29.9)	345.1 (28.5)	0.428	1.07 (0.90–1.28)
TGG	180.3 (14.3)	203.9 (16.9)	0.070	0.82 (0.66–1.02)	293.4 (23.9)	203.9 (16.9)	<0.001	1.56 (1.27–1.90)
Other^*^	2.0 (0.2)	6.0 (0.5)	?	?	8.0 (0.6)	6 (0.5)	?	?
***TNF***								
CACGG	189.0 (15.6)	242.9 (19.2)	0.018	0.78 (0.63–0.96)	271.3 (22.1)	189.0 (15.6)	<0.001	1.54 (1.25–1.89)
CCCGA	—				53.3 (4.3)	22.0 (1.8)	<0.001	2.46 (1.49–4.07)
TCCAG	95.5 (7.9)	93.9 (7.4)	0.666	1.07 (0.79–1.44)	25.2 (2.1)	95.5 (7.9)	<0.001	0.25 (0.16–0.38)
TCCGG	716.6 (59.2)	718.1 (56.8)	0.221	1.11 (0.94–1.30)	591.2 (48.2)	716.6 (59.2)	<0.001	0.64 (0.55–0.75)
TCTGG	181.5 (0.2)	180.0 (14.2)	0.595	1.06 (0.85–1.33)	277.8 (22.7)	181.5 (15.0)	<0.001	1.67 (1.35–2.05)
Other^*^	27.6 (2.3)	29.1 (2.3)			7.2 (0.5)	5.6 (0.5)		

CI, confidence interval; OR, odds ratio.

^*^Haplotypes with frequency <0.03 were pooled into this category.

To validate the aforementioned results, we performed an independent study in a Chinese Tibetan population (Tables 3 and 4). Among the three SNPs analyzed in *IL1B*, the G allele (P = 0.023) and GG genotype (P = 0.028) of rs16944 were more prevalent in controls than in TB patients. No significant difference was identified in the distributions of *IL6* genotypes in TB and control groups. In *TNF*, the rs1799964 C allele (*P* = 0.011) and CT genotype (*P* = 0.004) as well as the rs1800630 A allele (*P* = 0.005) and CA genotype (*P* = 0.002) increased risk of TB. SNPs in *TNF* were also associated with TB under other genetic models: rs1799964 (dominant: *P = *0.004), rs1800630 (dominant: *P* = 0.017), rs1799724 (dominant: *P* < 0.001; recessive: *P* = 0.003) and rs1800629 (dominant: *P* = 0.038). The LD between the SNPs is shown in Fig. 1. In the haplotype analysis, a total of three haplotypes in *IL1B*, two in *IL6* and five in *TNF* were found to be associated with TB (Table 5).

### Examination of smoking-specific effects with TB

We also determined whether genetic determinants of TB were smoking-dependent in the Chinese Han population by performing an allele-smoking interaction analysis. As shown in Table 6, *TNF* rs1800630 (*P* = 0.026) was associated with TB in the non-smoking group, but not in the smoking group. In addition, 5 SNPs (*IL1B*, rs16944: *P* < 0.001; *IL6*, rs1800795: *P* < 0.001; rs2069837: *P* < 0.001; *TNF*, rs1800629: *P* = 0.001; rs361525: *P* < 0.001) within the three genes were associated with smoking TB.

**Table 6:** Association of cytokine genes with TB stratified by smoking status.

Gene/SNPs	Genetic model	Non-smoking		Genetic model	Smoking	
		*P* ^#^	OR^#^ (95% CI)		*P* ^#^	OR^#^ (95% CI)
*IL1B*	Allele			Allele		
rs1143634G > A		0.166	1.63 (0.82–3.23)		0.986	—
rs16944G > A		0.883	1.02 (0.83–1.24)		<0.001	153.50 (37.79–623.61)
rs1143623C > G		0.430	1.08 (0.89–1.32)		0.451	1.12 (0.84–1.50)
*IL6*	Allele			Allele		
rs17147230A > T		0.565	0.94 (0.77–1.15)		0.130	1.26 (0.94–1.69)
rs1800795G > C		0.226	0.26 (0.031–2.28)		<0.001	0.001 (0–0.10)
rs2069837A > G		0.046	0.77 (0.59–1.00)		<0.001	59.03 (8.18–426.17)
*TNF*	Allele			Allele		
rs1799964T > C		0.017	1.35 (1.05–1.73)		0.427	0.86 (0.60–1.24)
rs1800630C > A		0.026	1.34 (1.04–1.72)		0.653	0.92 (0.62–1.34)
rs1799724C > T		0.804	0.97 (0.73–1.27)		0.654	0.91 (0.60–1.38)
rs1800629G > A		0.156	0.76 (0.52–1.11)		0.001	0.48 (0.30–0.75)
rs361525G > A		0.961	1.02 (0.47–2.21)		<0.001	0.26 (0.13–0.56)

SNPs, single nucleotide polymorphisms; CI, confidence interval; OR, odds ratio.

^#^Adjusted by age and sex.

## Discussion

Understanding the genetic factors underlying TB has attracted increasing attention. In this study, we analyzed the association between polymorphisms in three cytokine genes and TB in two independent studies. We found the *IL1B* rs16944 was associated with TB susceptibility in the two studies. Significant associations were also found for both rs1799964 and rs1800630 in *TNF* with TB, which were validated in the Tibetan population.

IL1B is a member of the IL1 cytokine family, which has an important role in the initiation and propagation of immune and inflammatory reactions. IL1B is a typical proinflammatory cytokine which can promote both local and systemic responses^41^. It was shown that alveolar macrophages from active TB subjects secreted high levels of IL1B^42^. Additionally, the production of this cytokine was associated with severity of human TB^43^. In addition, *IL1B* was reported to play an important role in the pathogenesis of TB in mice and human subjects^44,45^. rs1143634 and rs16944 are functional SNPs in *IL1B*. rs16944 has been associated with some diseases such as esophageal cancer^46^, inflammatory bowel disease^47^ and schizophrenia. However, rs16944 was variably associated with TB among different populations. One report in a small cohort from China suggested no association between the SNP and TB disease^48^. A similar study conducted in Kazakhstan did not show a significant association between the risk of TB and rs16944^49^. Awomoyi *et al*. reported that rs16944 both heterozygosity and homozygosity were a protective factor for TB in Gambian^50^, in accordance with our results in the Chinese Han population. However, in our validation cohort, we found that rs16944 G allele and GG genotype instead of AA/GA were risk factors for TB. It was reported that rs16944 regulates IL1B levels and the homozygosity TT genotype had the highest expression of *IL-1β*^34,38^. Higher IL-1β levels could also lead to the progressive immunopathology during *M*. *TB* infection, which may result in TB progression in human^45^. Combined with the information above, we speculate that rs16944 SNP may influence immune reaction caused by *M*. *TB* infection by increasing IL1B levels and RNA expression. Two other SNPs (rs1143634 and rs1143623) were not associated with TB in the two cohorts. This result was the same as previous studies that were conducted in various populations^16,51–53^.

IL6 is involved in the immune response, inflammation, coagulation, cell differentiation and tumorigenesis. High IL6 levels were related to inflammatory diseases such as rheumatoid arthritis^54^. In local inflammatory reactions, IL6 induces chemotaxis to mononuclear phagocytes^55^. When infected with *M*. *TB*, impaired bronchial epithelial cells activate mononuclear cells and lymphocytes to produce IL6, which then produces numerous antigen immune responses^56^. IL6 promotes IFNγ induction in early inflammatory responses and the important role of IL6 in TB susceptibility has been identified by *IL6* knock-out mice^24^. A recent study in the Chinese Tibetan population showed an association between the rs2069837 G allele and TB^57^. However, there were no studies have been researched the association between rs2069837 polymorphism and its function. Our results in the Han population are consistent with He *et al*.; although the observation failed to validate in our Tibetan cohort. Those inconsistent results could be explained by differences in the original of the samples. Subjects in our validation cohort were derived from the Aba Tibetan Autonomous Prefecture, and their subjects were recruited from the Tibet Autonomous Region. We also demonstrated that the other two SNPs (rs17147230 and rs1800795) were not associated with TB in the Han and Tibetan populations. More studies are warranted to validate these results in the future.

The response to *M*. *TB* infection is characterized by a strong immunocyte-mediated immune response, with the induction of *TNF* gene expression. TNF-alpha is important in the control of *M*. *TB* infection, and latent infection subjects with blocked *TNF* production can rapidly reactivate^58^. TNF-alpha not only plays a critical role in the immune response to TB but also participates in granuloma formation^59^. In our initial study, we demonstrated that rs1799964 (allele C and heterozygote CT) and rs1800630 (allele A and heterozygote CA) were protective factors against TB. The same results were also detected in our validation cohort, which strongly suggested that *TNF* was a causal gene for TB susceptibility. However, the r^2^ between rs1799964 and rs1800630 was 0.87 and 0.81, respectively in the Han and Tibetan cohort, signifying that the association of TB with rs1800630 may be due to its LD with rs1799964 or vice versa. Studies have demonstrated that the rs1799964 and rs1800630 could increase the *TNF* RNA expression and secretion of the TNF-alpha protein^31,36,39^. In the present study, the same genotypes were associated with TB disease. Therefore, we speculate that these two SNPs may control the progression of TB disease by increasing the RNA expression and cytokine levels of *TNF*. In addition, overexpression of TNF-alpha has been implicated in the immunopathology of TB, such as tissue necrotising reactions which are important for the transmission of *M*. *TB*^60,61^. However, previous study proposed that other genes polymorphisms were also found to control the production of the cytokines included in our study^62^. Thus, a limitation of our study was that we did not perform functional validation of the significant SNPs.

A published study in the Chinese Uygur population observed that rs1799964 was associated with TB drug-resistance whereas it was not related to TB susceptibility^63^. They also found the rs1800630 AA + CA genotype was a risk factor for TB. The significant association of rs1799724 and rs1800629 with TB in our Tibetan cohort was the same as a previous study^63–65^. A published meta-analysis suggested that rs361525 was not associated with TB^66^ and our study in both the Han and Tibetan populations also reports the same result. Since some of our significant findings were only detected in dominant/recessive models calculated by logistic regressions, correction for multiple comparisons such as Bonferroni may be appropriate to reduce a type I error in the data analysis. However, limiting the type I error may simultaneously increase the type II error^67^. In addition, the primary finding in this study was the significant association between *TNF* polymorphisms and TB disease in allele/genotype models and the association did not change in dominant/recessive models. What’s more, this association was validated in the Tibetan population, which suggested that this finding was not due to chance. Moreover, the rs1800630 showed the same effect on TB disease in previous studies as ours (Table S2). Therefore, Bonferroni correction may have limited utility in our study.

As mentioned above, all of the three cytokine genes were associated with TB in the initial study. However, only *TNF* polymorphisms rs1799964 and rs1800630 were fully validated under the allele/genotype model in the Tibetan group. Thus, we speculated that *TNF* has a more critical role in TB risk than other two cytokines. Meanwhile, the contradictory results in our study are also worth consideration. These discrepant results may be due to the differences in genetic and environmental factors between the Han and Tibetan populations. Firstly, the MAF and genetic background between the two cohorts were different, which may have caused the different genetic association results. Secondly, many lifestyle factors were different between the two populations. Most Tibetans live in the plateau area and their staple foods are barley, beef and butter. However, the effect of the environment on our association results is unknown.

In the Tibetan cohort, the HWE test for rs1800630 was *P* = 0.024 in the control group. This deviation was not found in the case group. To detect if there were any genotyping errors, we repeated genotyping in 5% of the samples, and the results remained the same. The deviation from HWE could be explained by natural selection. In 1949, geneticist JBS Haldane proposed that infectious diseases have been the primary means of natural selection during the past 5000 years^68^.

Stratified analyses were performed on the 11 SNPs based on smoking status at TB onset in the Chinese Han population (subjects were divided into smoking and non-smoking). Interestingly, we found that the associations of *IL1B* and *IL6* polymorphisms were more pronounced among smokers, which was similar to a previous study of TB^69^. Besides, SNPs within *TNF* revealed significant differences in allele frequencies between TB and controls in both smoking and non-smoking groups. Smoking is likely to be a risk factor for TB progression^70^. Our results further underline the critical role of smoking at TB onset as an important factor to consider in future TB association studies to reduce TB phenotypic heterogeneity.

In our study, a total of three haplotypes in *IL1B*, two in *IL6* and five in *TNF* were strongly associated with TB in the Tibetan population. In addition, one haplotype in *TNF* showed significant differences between cases and control in the Han population. However, the results of haplotypes in the two cohorts were inconsistent. Since haplotype-based methods were based on the association between a polymorphism and the ancestral haplotype^71^, we speculate the discrepant results are likely attributable to differences in demographic history^72^.

Nevertheless, several limitations should be addressed in this study. First, nearly all the OR we observed were <2, which suggested that the power of the study may be inadequate to detect a role of the three gene polymorphisms on TB. However, our sample size was larger than most of the previous genetic association studies (Table S1). Second, functional validation of the meaningful SNPs was not performed. Thus the mechanism underlying the genetic association is unknown.

## Conclusion

In summary, our study demonstrated that one SNP (rs16944) in *IL1B* and two SNPs (rs1799964 and rs1800630) in *TNF* were associated with susceptibility to or protection against TB development in our two studies. In addition, *IL6* rs2069837 was a risk factor for TB in the Chinese population. Furthermore, *TNF* rs1799724 and rs1800629 were associated with TB in the Tibetan cohort. Our study could enhance the understanding of TB pathogenesis for clinicians and may improve the diagnosis of TB in the future. Further studies in other ethnic groups are needed to fully validate these results to disclose the potential function of these SNPs.

## Methods

### Study population

In the initial cohort, a total of 636 TB patients and 608 healthy controls were included from the West China Hospital of Sichuan University. An independent validation cohort including 613 TB patients and 603 healthy was enrolled from the Aba Tibetan Autonomous Prefecture. TB cases were diagnosed by two independent experienced respiratory physicians based on sputum smear tests, sputum culture, clinical symptom, radiological, histological pathologic examination and positive response to anti-TB therapy. Healthy controls were who had no TB-related symptoms, previous of history of TB and chest x-ray signs of disease. However, most of the participants lack the detection of Tuberculin Skin Test (TST) and Interferon Gamma Release Assay (IGRA).

Participants with HIV, hepatitis B and C, cancer and immune-related diseases were excluded from this study. All of the study subjects were unrelated. Specialized nurses drew 2–5 mL of blood from each study participant into tubes containing EDTA. Blood sample was then preserved in an −80 °C freezer for further DNA extraction and genotyping. Written informed consent was obtained from each participant. All experimental procedures were done in a BSL2 laboratory. This study was approved by the Ethics Committees of the West China Hospital of Sichuan University and the People’s Hospital of the Aba Tibetan Autonomous Prefecture. All experimental procedures were conducted in accordance with the approved guidelines and regulations and the Declaration of Helsinki.

### SNP selection and genotyping

Candidate SNPs for this study were selected according to the literature review of previous studies and in silico functional prediction from the NIH FuncPred website (https://snpinfo.niehs.nih.gov/snpinfo/snpfunc.html). SNPs were selected if they were reported to be associated with disease and/or predicted to have effects on function. Genomic DNA was isolated from 250 μL of blood using a genomic DNA purification kit according to the manufacturer’s instructions (Axygen Scientific Inc, Union City, CA, USA). SNPs were genotyped using the improved multiplex ligase detection reaction (iMLDR), with technical support from the Shanghai Genesky Biotechnology Company. A subgroup of 5% of the samples was repeated by iMLDR to confirm reproducibility.

### Statistical analysis

Hardy-Weinberg equilibrium (HWE) proportion tests were used to evaluate the quality of the genotype data. Continuous variables were calculated by Student t-test and categorical variables were assessed by χ^2^-test in our study. The distribution of genotypes/alleles between cases and controls were compared by computing the odds ratio (OR) and 95% confidence intervals (95% CI) using logistic regression analyses. ORs were defined with respect to the case groups i.e. ORs >1 represent increased risk of TB. Linkage disequilibrium (LD) measure r^2^ was calculated and haplotype blocks were estimated using the SHEsis online software platform (http://analysis.bio-x.cn). The power of our study design was assessed by using the Power and Sample Size Calculation software^73^. We calculated the power for each SNPs under an allelic model as described previously^74^. The frequency of each SNP in the two cohorts was sufficient to provide reasonable power (>80%) to detect an allele/genotype effect with OR 2.0 or above (Table 7). Data were considered statistically significant if *P* < 0.05. All analyses were performed utilizing the Statistical Package for the Social Sciences (SPSS, SPSS Inc., Chicago, IL, USA), version 19.0.

**Table 7:** Power of the study with different odds ratios under the allele model.

SNPs	Power in Han				Power in Tibetan			
	MAF	OR = 2	OR = 3	OR = 4	MAF	OR = 2	OR = 3	OR = 4
***IL1B***								
rs1143634	0.02	0.81	0.99	1	0.03	0.92	1	1
rs16944	0.48	1	1	1	0.42	1	1	1
rs1143623	0.41	1	1	1	0.48	1	1	1
***IL6***								
rs17147230	0.44	1	1	1	0.48	1	1	1
rs1800795	0.002	0.15	0.34	0.55	0.002	0.15	0.34	0.55
rs2069837	0.17	1	1		0.26	1	1	1
***TNF***								
rs1799964	0.17	1	1	1	0.22	1	1	1
rs1800630	0.16	1	1	1	0.18	1	1	1
rs1799724	0.14	1	1	1	0.16	1	1	1
rs1800629	0.07	0.99	1	1	0.02	0.80	0.99	1
rs361525	0.02	0.81	0.99	1	0.04	0.97	1	1

SNP, single nucleotide polymorphism; TB, tuberculosis; OR, odds ratio; MAF, minor allele frequency.

## Supplementary information

Supplementary Dataset 1

Supplementary Dataset 1

## Data Availability

The data from this study are available online (https://figshare.com/s/33c072e600546dc1ba14).
