# Supplementary material for: Polymorphisms of cytokine genes and tuberculosis in two independent studies
Source: Sci Rep. 2019 Feb 21;9:2507. doi: 10.1038/s41598-019-39249-4 (PMC6385216; doi:10.1038/s41598-019-39249-4)
Supplement: Supplementary file 1 — Supplementary Dataset 1 [file 41598_2019_39249_MOESM1_ESM.docx]

| Gene/polymorphisms | Genetic model | Han population* | |  | Tibetan population* | |  | Published data | | |
| --- | --- | --- | --- | --- | --- | --- | --- | --- | --- | --- |
|  |  | *P* | OR(95%CI) |  | *P* | OR(95%CI) |  | *P* | OR(95%CI) | reference |
| *IL1B* |  |  |  |  |  |  |  |  |  |  |
| rs1143634 | Homozygote | - |  |  | - |  |  | 0.780 | - | ^1^ |
|  | Allele | 0.183 | 1.50(0.83-2.73) |  | 0.133 | 1.41(0.90-2.21) |  | 0.440 | 0.87(0.61-1.24) | ^2^ |
|  | Heterozygote | 0.094 | 1.71(0.91-3.19) |  | 0.184 | 1.37(0.86-2.16) |  | 0.500 | 0.84 (0.48-1.47) | ^3^ |
|  | Allele |  |  |  |  |  |  | 0.395 | 0.40(0.55–1.30) | ^4^ |
|  | Allele |  |  |  |  |  |  | 0.049 |  | ^5^ |
| rs16944 | Heterozygote | 0.463 | 0.90(0.68-1.19) |  | 0.105 | 0.81(0.62-1.05) |  | 0.027 | 0.66(0.45.0.97) | ^2^ |
|  | Heterozygote |  |  |  |  |  |  | 0.682 | 0.98(0.88–1.08) | ^6^ |
| rs1143623 | Heterozygote | 0.709 | 1.05(0.82-1.35) |  | 0.731 | 1.05(0.80-1.38) |  | 0.673 | 0.98(0.88–1.09) | ^6^ |
| *IL6* |  |  |  |  |  |  |  |  |  |  |
| rs17147230 | Allele | 0.328 | 0.92(0.79-1.08) |  | 0.344 | 0.93(0.79-1.09) |  | 0.772 | 1.03(0.83–1.28) | ^7^ |
| rs1800795 | Dominant | 0.161 | 0.32(0.06-1.58) |  | 0.121 | 3.47(0.72-16.79) |  | 0.188 | 0.69(0.58-0.83) | ^8^ |
| rs2069837 | Dominant | 0.044 | 0.78(0.62-0.99) |  | 0.820 | 1.03(0.82-1.29) |  | <0.001 | 1.97(1.53–2.55) | ^9^ |
| *TNF* |  |  |  |  |  |  |  |  |  |  |
| rs1799964 | Allele | 0.011 | 1.30(1.06-1.58) |  | 0.011 | 1.27(1.06-1.53) |  | >0.05 | 0.99(0.75-1.32) | ^10^ |
| rs1800630 | Allele | 0.020 | 1.28(1.04-1.58) |  | 0.005 | 1.33(1.09-1.62) |  | <0.05 | 2.53(1.73-3.69) | ^10^ |
|  | Allele |  |  |  |  |  |  | 0.007 | 2.42(1.28-4.59) | ^11^ |
| rs1799724 | Allele | 0.499 | 0.93(0.74-1.16) |  | <0.001 | 1.62(1.32-1.99) |  | 0.0009 | 0.67(0.52–0.85) | ^11^ |
|  | Allele |  |  |  |  |  |  | >0.05 | 1.05(0.78-1.42) | ^10^ |
|  | Allele |  |  |  |  |  |  | 0.522 | - | ^12^ |
| rs1800629 | Allele | 0.545 | 0.91(0.68-1.23) |  | 0.056 | 0.62(0.38-1.01) |  | 0.005 | 2.88(8–4.77) | ^13^ |
|  | Allele |  |  |  |  |  |  | 0.024 | 1.96(1.04-3.71) | ^14^ |
|  | Allele |  |  |  |  |  |  | >0.05 | 1.29(0.99-1.69) | ^10^ |
|  | Allele |  |  |  |  |  |  | 0.540 | 0.79(0.37–1.68) | ^15^ |
|  | Allele |  |  |  |  |  |  | 0.814 | 1.05(0.68-1.60) | ^16^ |
|  | Allele |  |  |  |  |  |  | >0.05 | - | ^17^ |
| rs361525 | Allele | 0.908 | 1.03(0.59-1.81) |  | 0.740 | 1.07(0.73-1.57) |  | 0.589 | 0.82(0.39–1.70) | ^15^ |
|  | Allele |  |  |  |  |  |  | >0.05 | 1.48(0.770-2.86) | ^10^ |
|  | Allele |  |  |  |  |  |  | 0.430 | - | ^12^ |

Table S2 Comparison of the findings between our study and published studies.

Abbreviation: PTB, pulmonary tuberculosis; CI, confidence interval; OR, odds ratio;

*Resutls in our study

1 Delgado, J. C., Baena, A., Thim, S. & Goldfeld, A. E. Ethnic-specific genetic associations with pulmonary tuberculosis. *J Infect Dis* **186**, 1463-1468, doi:10.1086/344891 (2002).

2 Awomoyi, A. A. *et al.* Polymorphism in IL1B: IL1B-511 association with tuberculosis and decreased lipopolysaccharide-induced IL-1beta in IFN-gamma primed ex-vivo whole blood assay. *Journal of endotoxin research* **11**, 281-286, doi:10.1179/096805105X58706 (2005).

3 Naslednikova, I. O. *et al.* Allelic polymorphism of cytokine genes during pulmonary tuberculosis. *Bull Exp Biol Med* **148**, 175-180 (2009).

4 Meenakshi, P. *et al.* Association of IL-1beta +3954 C/T and IL-10-1082 G/A cytokine gene polymorphisms with susceptibility to tuberculosis. *Scand J Immunol* **78**, 92-97, doi:10.1111/sji.12055 (2013).

5 Motsinger-Reif, A. A. *et al.* Polymorphisms in IL-1beta, vitamin D receptor Fok1, and Toll-like receptor 2 are associated with extrapulmonary tuberculosis. *BMC medical genetics* **11**, 37, doi:10.1186/1471-2350-11-37 (2010).

6 Zhang, G. *et al.* Allele-specific induction of IL-1β expression by C/EBPβ and PU. 1 contributes to increased tuberculosis susceptibility. *PLoS pathogens* **10**, e1004426 (2014).

7 Shen, C. *et al.* A 3'UTR polymorphism of IL-6R is associated with Chinese pediatric tuberculosis. *Biomed Res Int* **2014**, 483759, doi:10.1155/2014/483759 (2014).

8 Wang, H. *et al.* Association between the IL-6 gene polymorphism and tuberculosis risk: a meta-analysis. *Infect Drug Resist* **10**, 445-454, doi:10.2147/IDR.S144296 (2017).

9 He, S. *et al.* Association of IL4, IL6, and IL10 polymorphisms with pulmonary tuberculosis in a Tibetan Chinese population. *Oncotarget* **9**, 16418-16426 (2018).

10 Li, Q. *et al.* The single nucleotide polymorphisms in TNF-α promoter are associated with susceptibility and clinical features of pulmonary tuberculosis in Chinese Uygurs. *International journal of clinical and experimental medicine* **10**, 11596-11605 (2017).

11 Ma, M. J. *et al.* Toll-like receptors, tumor necrosis factor-alpha, and interleukin-10 gene polymorphisms in risk of pulmonary tuberculosis and disease severity. *Hum Immunol* **71**, 1005-1010, doi:10.1016/j.humimm.2010.07.009 (2010).

12 Kusuhara, K., Yamamoto, K., Okada, K., Mizuno, Y. & Hara, T. Association of IL12RB1 polymorphisms with susceptibility to and severity of tuberculosis in Japanese: a gene-based association analysis of 21 candidate genes. *International journal of immunogenetics* **34**, 35-44, doi:10.1111/j.1744-313X.2007.00653.x (2007).

13 Mabunda, N. *et al.* Gene polymorphisms in patients with pulmonary tuberculosis from Mozambique. *Molecular biology reports* **42**, 71-76, doi:10.1007/s11033-014-3741-1 (2015).

14 Ben-Selma, W., Harizi, H. & Boukadida, J. Association of TNF-alpha and IL-10 polymorphisms with tuberculosis in Tunisian populations. *Microbes Infect* **13**, 837-843, doi:10.1016/j.micinf.2011.04.009 (2011).

15 Zhou, Y. *et al.* Polymorphisms in the SP110 and TNF-alpha Gene and Susceptibility to Pulmonary and Spinal Tuberculosis among Southern Chinese Population. *Dis Markers* **2017**, 4590235, doi:10.1155/2017/4590235 (2017).

16 Caliskan, T. *et al.* ASSOCIATION BETWEEN IFN-γ+ 874A/T, TNF-α-308G/A AND IL-12Rβ2–237C/T GENE POLYMORPHISMS AND SUSCEPTIBILITY TO PULMONARY TUBERCULOSIS IN A TURKISH POPULATION. *Acta Medica* **31**, 1291 (2015).

17 Sivangala, R. *et al.* Association of cytokine gene polymorphisms in patients with tuberculosis and their household contacts. *Scand J Immunol* **79**, 197-205, doi:10.1111/sji.12136 (2014).
